# Technology Integration Methods for Bi-directional Brain-computer Interfaces and XR-based Interventions

**DOI:** 10.1109/SMC42975.2020.9282993

**Published:** 2020-12-14

**Authors:** Kei Landin, Moaad Benjaber, Fawad Jamshed, Charlotte Stagg, Timothy Denison

**Affiliations:** MRC Brain Network Dynamics Unit, University of Oxford, Oxford, UK; MRC Brain Network Dynamics Unit, University of Oxford, Oxford, UK; MRC Brain Network Dynamics Unit, University of Oxford, Oxford, UK; Wellcome Centre for Integrative Neuroimaging, University of Oxford, Oxford, UK; MRC Brain Network Dynamics Unit, University of Oxford, Oxford, UK

**Keywords:** brain-computer interfaces, DyNeuMo, DBStim, Summit RC+S, virtual reality, XR, MR, neurorehabilitation

## Abstract

Brain stimulation therapies have been established as effective treatments for Parkinson’s disease, essential tremor, and epilepsy, as well as having high diagnostic and therapeutic potential in a wide range of neurological and psychiatric conditions. Novel interventions such as extended reality (XR), video games and exergames that can improve physiological and cognitive functioning are also emerging as targets for therapeutic and rehabilitative treatments. Previous studies have proposed specific applications involving non-invasive brain stimulation (NIBS) and virtual environments, but to date these have been uni-directional and restricted to specific applications or proprietary hardware. Here, we describe technology integration methods that enable invasive and non-invasive brain stimulation devices to interface with a cross-platform game engine and development platform for creating bi-directional brain-computer interfaces (BCI) and XR-based interventions. Furthermore, we present a highly-modifiable software framework and methods for integrating deep brain stimulation (DBS) in 2D, 3D, virtual and mixed reality applications, as well as extensible applications for BCI integration in wireless systems. The source code and integrated brain stimulation applications are available online at https://github.com/oxfordbioelectronics/brain-stim-game

## I Introduction

Neurotechnologies and creative innovations in extended reality (XR) and game-based interventions are emerging at a rapid pace [1]–[3], opening up an unexplored field of translational research. Brain stimulation therapies and virtual environments including XR, application software, and video games, share many potential applications in therapeutic and rehabilitative medicine [4]–[7]. Deep brain stimulation (DBS), and non-invasive brain stimulation (NIBS) including transcranial magnetic stimulation (TMS) and transcranial electrical stimulation (tES) are steadily evolving, paving the way for new potential applications in medicine and clinical neuroscience [8]–[11]. For example, by modulating neural activity in key brain structures, DBS can help alleviate symptoms of common movement disorders [8], [12]. More broadly, video games and “exergames” which combine physical activity, have demonstrated the ability to attenuate physical and mental deterioration [13], [14], promote physiological and cognitive functioning in clinical and non-clinical populations [15], [16], show positive implications for executive function and global cognition, and help mitigate symptoms associated with Alzheimer’s disease and mild cognitive impairment (MCI) [5], [17], [18].

XR-based interventions also present potential utility for functional neurosurgery patients [19], providing a controlled environment and a reported increased level of understanding, comfort, and satisfaction among patients [20]. The untapped potential of combining virtual environments and brain stimulation has led to an influx of new research, creating a new technological niche [21]–[23]. Game-based interventions including exergames and XR applications have been integrated with electroencephalography (EEG) and transcranial alternating current stimulation (tACS) for recording and monitoring purposes [24]–[26].

Sensing systems such as brain-computer interfaces, in particular EEG-based BCI, have been applied to neurofeedback applications, which aim to train the user to self-modulate brain function [27], [28]. Nevertheless, attempts to integrate brain stimulation and virtual environments remain few [21], [24], [29], and are often uni-directional or require proprietary hardware, limiting their potential use and applications [24], [29], [30]. The deployment of a generalized approach to integrating neurotechnologies could help expand research efforts in brain-computer interfaces and XR-based interventions.

In this paper, we present a versatile framework and integration methods that enable brain sensing and stimulation devices to interface with application software. We also present prototype applications that integrate DBS, examples of brain stimulation devices that are able to interface with the development platform Unity, as well as tools that can be used for developing BCI and XR applications. The source code is available online and can be customized to interface with most current neurotechnologies in developing application software for further research.

## II System Overview

### A General Design Environment

Brain stimulation devices including non-invasive brain stimulation (NIBS) and deep brain stimulation (DBS) may support sending and receiving data to application software by interfacing to a development platform such as Unity or Unreal Engine via an intermediate microcontroller. Computer to microcontroller interfacing is mediated by a serial communication connection over USB. For serial communication, the .NET Framework by Microsoft can be used. Unity supports a comparatively large number of .NET profiles. These include API compatibility for .NET Standard 2.0 and .NET 4.X, with .NET 5 scheduled for release. Serial communication in Unity commonly use native code that implements either I/O or .NET Framework (System.IO.Ports), depending on the editor version used for development and project dependencies.

The applications presented in this paper were developed in Unity using a serial controller script for interfacing directly with a brain stimulation device. The serial controller creates a thread in which it polls a USB serial port, storing all received data. Depending on the configuration of Unity, the serial controller can be used to configure a callback function each time data is available, for example calling *ReadSerialMessage(),* or *SendSerialMessage().* When developing application software in Unity, a general approach may involve attaching a *SerialController* script to a game object for initiating the serial port connection when added to a scene. Serial communication in Unity may provide a general method for enabling bi-directional communication and implementation of custom communication protocols for creating a brain-computer interface. In principle, this method may be used for simultaneously integrating multiple devices to application software and creating cross interfaces.

### B Scripting API

Unity is a C++ based application development framework with a primary object-oriented application programming interface (API) in C#. Prototyping and implementation of functionality are managed using the graphical Unity interface and editor, in addition to custom scripts. Scripts are essential to Unity development and can be used for controlling player input, managing game events and physics, creating animation and graphical effects, and implementing AI systems for non-player characters, to name a few. In addition, Unity provides support for managed plugins (.NET assemblies) and native plugins (C, C++, Objective-C) for calling functions from external libraries using Unity scripts. Dynamically-linked library (DLL) files can be used to expand the Unity scripting API.

The Unity API allows for an event-driven approach for interacting with objects and physics behaviors or events. Examples of this include non-kinematic and kinematic primitive colliders which can be assigned to game objects and referenced using custom scripts. Moreover, by using the Collision class (i.e. *OnTriggerEnter,* and *OnCollisionEnter*) in Unity, it is possible to add the *SerialController* script to any object in the game environment. For example, this allows the developer to set *OnCollisionEnter* to activate the *SerialController* when two objects collide. Likewise, *OnTriggerEnter* can be configured to activate the *SerialController* when the player progresses in the virtual environment, e.g. advancing beyond a checkpoint or reaching a new level. These fundamental game mechanics become powerful and versatile tools when integrating BCI technologies and neurostimulation devices.

## III Applications

### A DBStim, Transcranial Neurostimulator

The “DBStim” neurostimulator is a research tool designed to deliver high accuracy, biphasic constant current stimulation pulses to nervous tissue in various combinations of waveform, amplitude, pulse width, and frequency [31]. DBStim can be used to interface with externalized electrodes or as a transcranial stimulator. The stimulator uses a USB power and communication cable, and can interface with Unity via serial communication. DBStim has two output channels that connect to externalized deep brain stimulation leads implanted by surgeons near the selected brain targets to provide therapeutic stimulation for selected brain disorders such as Parkinson’s disease, essential tremor, epilepsy, and obsessive-compulsive disorder [8], [32]. Here, we present two applications that integrate deep brain stimulation using Unity and DBStim stimulator. The API provides an accessible framework for controlling brain stimulation parameters, including stimulation frequency, number of pulses, pulse and gap width, and channel polarity. Stimulation parameters can be adjusted within the Unity game environment, as well as programmed to change in response to specific event-based or reactive application software designs.

**Fig. 1:**
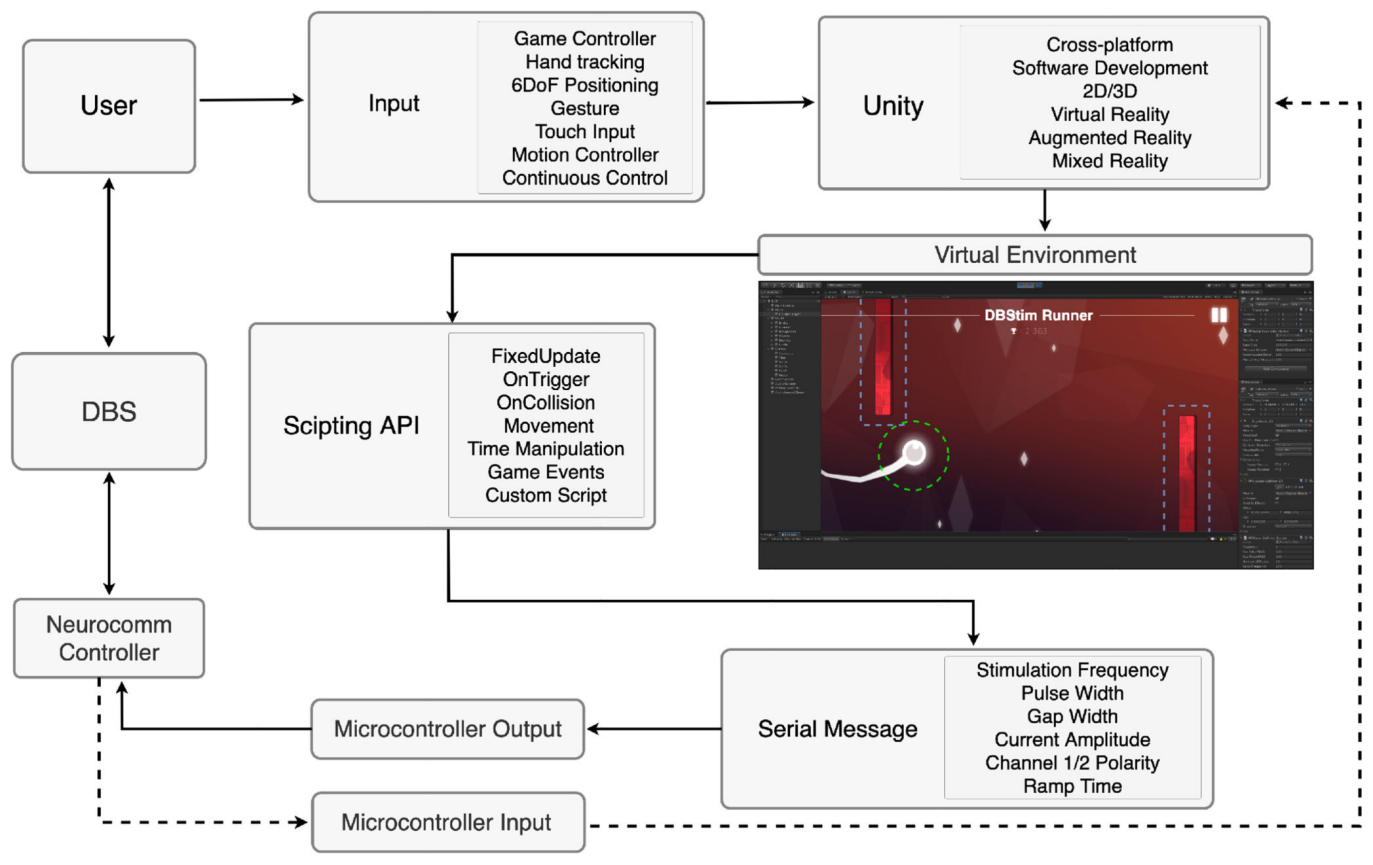
System overview showing multiple applications of bi-directional integrated brain stimulation mediated by a microcontroller using the development platform Unity. Examples of user input controls, platforms, game physics, and outgoing serial messages via microcontroller output for DBS are shown. The virtual environment depicts DBStim 2D integrating deep brain stimulation using serial communication and event-driven API calls. Stimulation parameters e.g. stimulation frequency, number of pulses, pulse and gap width, and channel polarity, can be adjusted using the Unity game environment. The Unity interface can provide clinicians and researchers easy access to stimulation parameter settings, as well as configurable real-time control in application software.

The first application is “DBStim 2D”, a runner-type 2D game that integrates brain stimulation API calls using collision boundaries for objects (e.g. player or non-player characters), and trigger zones (e.g. levels). The *DeepBrainStimCollider* script can be added to any game object, collider, or trigger, to send a serial command to DBStim stimulator for adjusting deep brain stimulation parameters as required. The stimulation pulses can be independently configured for multiple objects using the Unity interface and API (Fig. 1). In addition, stimulation can be configured using public variables to provide accessible modification of the DBStim parameters within the Unity interface and editor.

The second application is “DBStim MR”, which expands on the integration method used for DBStim 2D by applying brain stimulation to XR. XR is recently being used to assist patients with Alzheimer’s disease by using site-specific on-demand DBS for medial temporal lobe (MTL) for the enhancement of memory [33]. MTL, specifically the entorhinal region, transforms daily experience into lasting memories and is one of the first brain regions affected by Alzheimer’s disease [34]. DBS of the MTL enhances memory for spatial information when applied at the learning stage [33]. VR can also be used to present control task-specific stimuli such as navigational routes, memory tasks, or spatial learning tasks to evoke specific parts of the brain [35]. DBStim MR is a novel BCI that implements mixed reality pass-through vision, six degrees of freedom (6DoF) positioning, hand tracking, and deep brain stimulation. DBStim MR features interactive virtual objects, which can be configured to enable stimulation and adjust stimulation parameters using serial communication via Bluetooth Classic or Bluetooth Low Energy protocols. The integration methods provided by the DBStim applications may be applied by researchers and developers that require precise stimulation control and wireless connectivity for XR and game-based interventions.

### B DyNeuMo Mk-2 Implantable BCI

The Dynamic Neuro-Modulator (DyNeuMo) Mk-2 is a cranial-mount, investigational neurostimulation research tool. The DyNeuMo Mk-2 can provide adaptive deep brain stimulation based on changes in brain activities, which are sensed using the implanted DBS electrodes [36], [37]. The DyNeuMo Mk-2 interface is mediated by the Bioinduction Picostim API, providing a conduit for creating arbitrarily complex algorithms for research via the telemetry link. The DyNeuMo Mk-2 interfaces to the host computer via a handheld patient controller (Picon) that connects via USB. The interface allows for bi-directional communication between DyNeuMo Mk-2 and Unity. The API is imported as a plugin in Unity to enable DyNeuMo Mk-2 function calls using Unity scipts. The API calls can be used to activate or deactive stimulation, as well as alternating between up to eight different stimulation programs that are stored on the implanted device. Furthermore, the API enables real-time acquisition of neural data from the implanted DyNeuMo Mk-2 to Unity using the Stream class (System.IO). Here, we present two applications that integrate the DyNeuMo Mk-2 using Unity.

First, we present “DyNeuMo-2 Neurofeedback”, a beta oscillation-targeted neurofeedback trainer. Neurofeedback training has been investigated as a therapeutic target for Parkinson’s disease, anxiety, epilepsy, attention deficit/hyperactivity disorder (ADHD), and substance abuse. Sensorimotor rhythm neurofeedback training has been demonstrated to reduce MPTP-induced parkinsonian symptoms in nonhuman primates undergoing L-DOPA treatment [38]. In addition, neurofeedback training targeting beta band oscillations (13-30 Hz) in subthalamic nucleus (STN) have been investigated as a potential therapeutic target for PD in humans [27], [28].

**Fig. 2:**
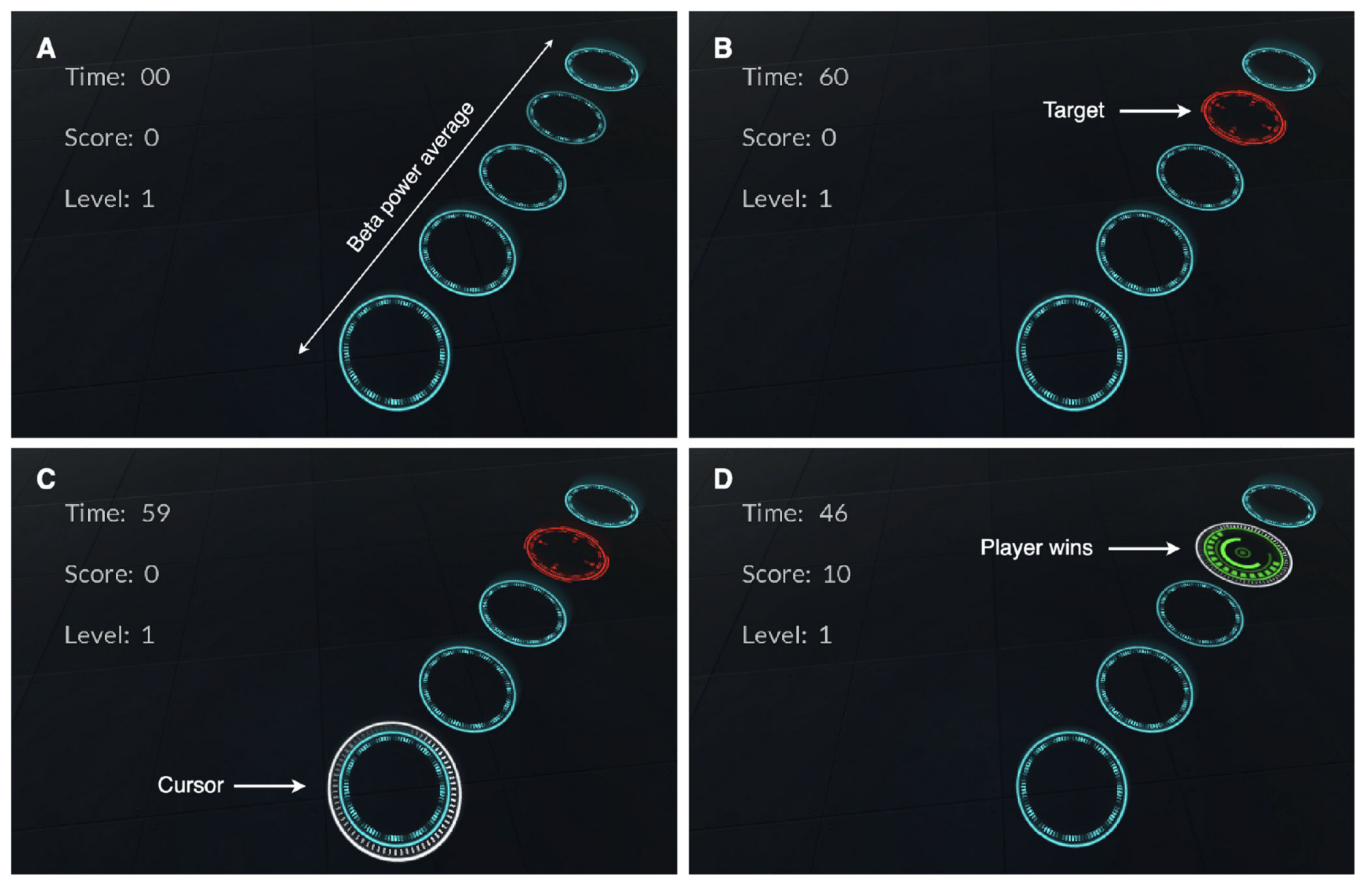
DyNeuMo-2 Neurofeedback is a beta oscillation-targeted neurofeedback trainer designed for PD patients to help increase the user’s ability to self-modulate beta band power from sensorimotor cortical areas as a potential therapeutic intervention for motor symptoms. (A) The game features five circles which correspond to different intervals of beta power, increasing vertically. (B) At game start a red target appears at a random location. (C) The player’s objective is to move a cursor (white circle) to the cued red target. The position of the cursor is updated in response to the real-time neural data which is streamed from the DyNeuMo Mk-2 to Unity. (D) The player can move the cursor by self-modulating their beta band power. The level is completed when the cursor overlaps with the red target. The DyNeuMo Mk-2 and Unity integration can be configured to enable adjustment of stimulation parameters in response to sensing data and game progression for closed-loop functionality.

DyNeuMo-2 Neurofeedback is a task-based game designed for PD patients to help increase the user’s ability to self-modulate beta band power from sensorimotor cortical areas as a potential therapeutic intervention for motor symptoms (Fig. 2). DyNeuMo-2 Neurofeedback builds on the approach of previous research adding adaptive deep brain stimulation and integration with Unity [27]. The application centres on the data streaming capabilities of the DyNeuMo Mk-2, providing real-time neural data from the player to control the position of a cursor. For controlling the cursor, the neural data must pass through a beta-filter. The data can then be rectified and averaged before being translated to update the cursor position in Unity. The game user interface consists of five circles that correspond to different intervals of beta band power, increasing vertically (Fig. 2). The player is tasked to move the cursor (white circle) towards a cued red target within a set time-limit. The position of the cursor is updated in response to the real-time neural data which is streamed from the DyNeuMo Mk-2 to Unity. The player can move the cursor up and down by self-modulating their beta band power. The level is completed when the cursor overlaps with the red target, i.e. when the beta band power average of the player matches that of the red target. After having completed a level, the player receives a score based on remaining time before proceeding to the next level. The application include multiple levels that alternative the position of the red target and time-limit. The DyNeuMo Mk-2 and Unity integration provides arbitrarily complex configuration of stimulation programs. This provides clinicians and researchers with the ability to configure the application in specific ways for individual patients, for example by activating specific stimulation programs or adjusting stimulation parameters in response to sensing data and/or game progression for closed-loop functionality.

**Fig. 3:**
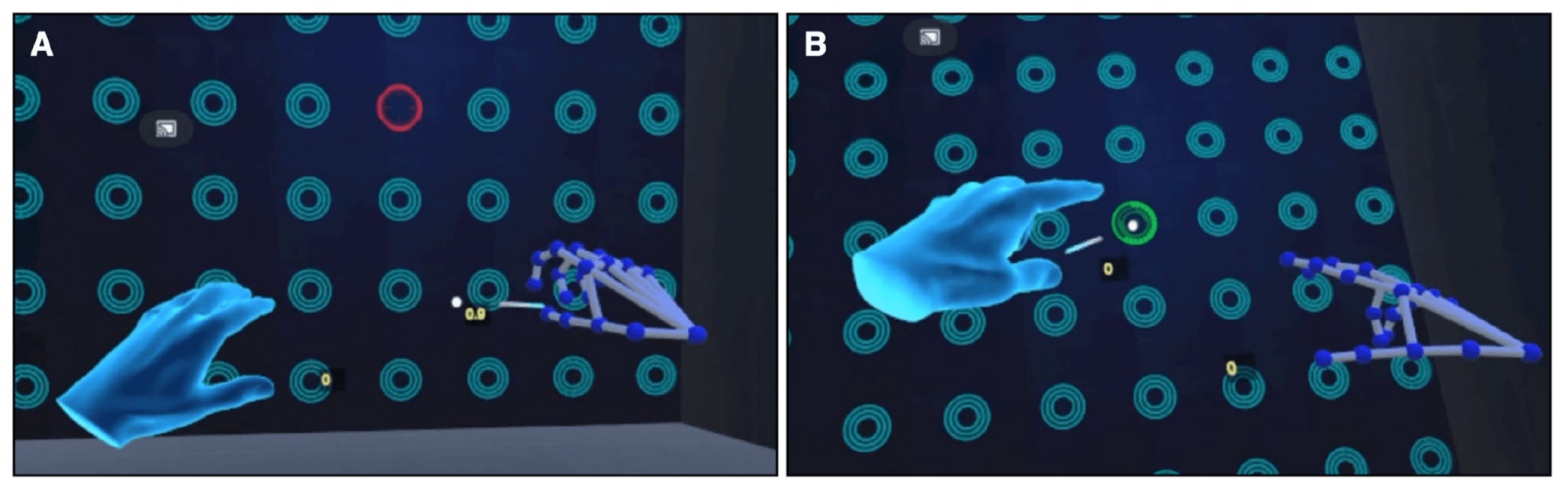
DyNeuMo-2 VR is a diagnostic and rehabilitative application for tremor patients. DyNeuMo-2 VR provides wireless PCVR, 21-point hand tracking, and integrated deep brain stimulation using the DyNeuMo Mk-2 and Unity. (A) The game consists of a 10x10 grid of blue circles facing the player in virtual reality. (B) The player’s objective is to point steadily at a red target which appears at random locations in the grid. DyNeuMo-2 VR is configured to alternate between up to eight different stimulation programs that are stored on the implanted device, depending on the current position and size of the red target. This allows for the different stimulation programs to be matched with the player’s performance using task time, accuracy, and hold duration data.

Second, we present “DyNeuMo-2 VR”, a diagnostic and rehabilitative tool designed for evaluating hand tremor and optimizing stimulation programs (Fig. 3). This application integrates deep brain stimulation in VR using the DyNeuMo Mk-2 and Unity. The application is built for wireless PCVR, providing compatibility for a wide range of VR systems including the HTC Vive and Oculus HMDs. DyNeuMo-2 VR implements the VIVE Sense SDK for advanced hand position and gesture recognition. The SDK provides 21-point finger tracking using the front-facing cameras for compatible devices such as Valve Index and HTC Vive HMDs. The DyNeuMo-2 VR game user interface consists of a 10x10 grid of blue circles facing the player in virtual reality. The player is tasked to steadily point with their finger at the cued red target using hand tracking (Fig. 3). The position and size of the red target change arbitrarily between tasks. To complete the task, the player should continuously hold position over the red target for a set period of time which is indicated by the target becoming green.

The integration of DyNeuMo Mk-2 provide precisely timed control of stimulation. The application is configured to alternate between up to eight different stimulation programs that are stored on the implanted device, depending on the current position and size of the cued red target. This allows for the different stimulation programs to be matched with the player’s performance using task time, accuracy, and hold duration data. When an optimal stimulation program has been identified, the tasks may be repeated using finer adjustments of individual stimulation parameters for further optimisation. This general method of integrating adaptive brain stimulation in task-based games could potentially be applied to a wide range of application software for developing custom user-tailored systems for patients. These applications are not limited to the DyNeuMo research system. Alternative investigational systems such as the Medtronic Summit RC+S might be used.

The Summit RC+S is a bi-directional neural interface that combines therapeutic neurostimulation with neural sensing. The Summit RC+S supports wireless stimulation control and communication of electrophysiological and motion sensing, as well as provides access to its API for potential Unity integration [39], [40]. For extensible applications, the Summit RC+S might provide a framework for the design and implementation of clinical applications that expand on the DyNeuMo Mk-2 API link and applications to facilitate exploration of generic closed-loop therapy algorithms.

## IV Discussion

The integration methods presented in this paper are configurable and provide potential for integrating various BCI technologies and neurostimulation devices. To date, there have been a wide range of applications integrating NIBS for rehabilitative or therapeutic interventions. Video games and exergames have been demonstrated to have a positive effect on basic activities of daily living (ADL) [41] and upper and lower extremity function in stroke patients [41], [42]. VR might help alleviate limitations in ADL due to anxiety, sense of deprivation and dependence, and hemiparesis [41]. tDCS and tACS have been shown to improve motor control in hemiparesis [43]. Stimulation of the lesioned or non-lesioned hemisphere can induce participation of its cells during movement [43]. Anodal stimulation of the ipsilesional hemisphere shows an increase in activity within the ipsilesional primary motor cortex in stroke patients [44] and enhances the functional benefits of rehabilitation [45]. tDCS has also shown potential in promoting long-term transfer of learning and cognitive enhancement when combined with game-based interventions. Stimulation of the dorsolateral prefrontal cortices improved performance in video games involving training learning and body movements [5]. Theta-gamma modulation using tACS has been demonstrated to significantly improve motor skill acquisition [46]. Moreover, Transcranial magnetic stimulation (TMS) shows potential as a powerful investigational device for high-resolution brain stimulation [47], and can be integrated with video games as an input controller [23]. These applications of NIBS show the broad potential and clinical implications of integrating brain stimulation in virtual environments.

The prototype applications presented in this paper provide examples of different types of brain stimulation devices interfacing with Unity. The DBStim stimulator applications demonstrate a general approach for integrating brain stimulation in Unity via serial communication, while providing examples of methods for controlling stimulation parameters in real-time using configurable in-game events. The DyNeuMo Mk-2 applications demonstrate the potential of integrating brain stimulation in Unity using both deep brain stimulation and real-time acquisition of neural data to create a closed-loop system. In addition, the Unity interface could provide a user-friendly graphical user interface (GUI), which would allow clinicians to communicate with medical devices for control of stimulation settings in virtual environments. This functionality could provide the ability for research sites to implement closed-loop therapy algorithms in clinical games and XR applications.

The integration methods for Unity leave room for improvement and further optimisation. The API link provides a general method for interfacing with devices with compatibility for serial communication to a host computer. Current methods are dependent on .NET Profile support which may differ depending on the Unity editor version. For interfacing with a new device, Unity may require considerable configuration and troubleshooting. At the start of the project, Unity was chosen over other developer platforms for its accessibility and potential to interface with external devices. For future research and development, Unreal Engine combined with UnrealCLR - a plugin which provides .NET Core and C# 9.0 compatibility - might provide a viable alternative to Unity as a developer platform for integrating BCI technologies and neurostimulation devices in application software. The integration of XR and devices can be greatly facilitated by API links. In the future, device researchers and manufacturers should consider supplying verified API libraries for third party developers. However, the final system will also be required to pass regulatory requirements for its intended purpose including appropriate quality measures such as 13485-software design controls.

## V Conclusion

Previous studies have established that XR and gamebased interventions and brain stimulation therapies share several clinical benefits and high therapeutic potential [5], [8], [32], [48], [49]. Research that focuses on combining neurotechnologies and virtual environments is rapidly expanding [1]-[3]. However, attempts to integrate brain stimulation are scarce, and are often uni-directional and inaccessible, providing low modifiability [24], [29], [30].

We present a novel framework and applications of integrating brain stimulation using a neurostimulator and Unity for bi-directional serial communication. In addition, we present four prototype applications that help demonstrate the high potential and applicability of integrating neurostimulators and BCI technologies in application software and XR-based interventions. The aim is to provide comprehensive tools and assets for developing bespoke in-house games and applications with integrated brain stimulation. The high versatility and multi-platform capabilities of this approach could enable a variety of brain-computer interfaces and neurostimulation devices to integrate with 2D, 3D, and extended reality applications, and provide a framework for future researchers to build on in developing clinical and non-clinical applications.

The source code and integrated brain stimulation implementations are available online at https://github.com/oxfordbioelectronics/brain-stim-game
